# Dysphagia in Multiple System Atrophy Does Not Correlate with Serum Neurofilament Light Chain and Glial Fibrillary Acidic Protein

**DOI:** 10.1002/mds.70092

**Published:** 2025-10-11

**Authors:** Martin Klietz, Katharina Pannewitz‐Makaj, Franziska Baacke, Florian Wegner, Matthias Höllerhage, Lea Krey, Lan Ye, Christoph Schrader, Günter U. Höglinger, Tobias Warnecke

**Affiliations:** ^1^ Department of Neurology Hannover Medical School Hannover Germany; ^2^ Department of Phoniatrics Hannover Medical School Hannover Germany; ^3^ Department of Neurology Ludwig‐Maximilians‐University of Munich Munich Germany; ^4^ German Center for Neurodegenerative Diseases (DZNE) Munich Germany; ^5^ Munich Cluster for Systems Neurology Munich Germany; ^6^ Department of Neurology and Neurorehabilitation Klinikum Osnabrueck Osnabrueck Germany

**Keywords:** multiple system atrophy, dysphagia, biomarker, neurofilament light chain, GFAP

Multiple system atrophy (MSA) is a rapid, progressive, neurodegenerative, atypical, parkinsonian syndrome resulting in disability and reduced life expectancy.^1^ Patients suffer from predominant cerebellar or parkinsonian symptoms combined with autonomic failure. Dysphagia is a very relevant and debilitating symptom of the disease. In recent years, several specific characteristics of dysphagia in MSA have been described. Patients with MSA suffer in particular from oral phase dysphagia but also show laryngeal involvement.^2^, ^3^ There seems to be no significant difference in the swallowing pattern between patients with cerebellar‐ or parkinsonian‐type MSA.^4^ However, these findings were detected with fiberoptic endoscopic evaluation of swallowing (FEES), a technique that is not widely available. The recent study by Katzdobler et al. reported a correlation of neurofilament light chain (NfL) and glial fibrillary acid protein (GFAP) in the cerebrospinal fluid/serum of MSA patients with clinical symptoms and even disease progression.^5^

This study investigated whether the serum biomarkers NfL and GFAP were associated with clinically relevant dysphagia and might thus be useful screening biomarkers for dysphagia in MSA.

Patients were diagnosed according to the Gilman criteria for MSA. A FEES investigation was performed by a certified investigator according to the FEES levodopa test protocol, and the FEES severity score of dysphagia was video analyzed by a blinded investigator.^6^ Serum NfL and GFAP were analyzed according to Katzdobler et al.^5^

For this study, 17 patients with MSA were recruited (10 parkinsonian type [MSA‐P], 7 cerebellar type [MSA‐C]). Seven possible MSA cases and 10 probable cases were included according to the Gilman criteria for MSA diagnosis. Demographics and clinical characteristics are presented in Table S1. FEES clinical predominant phenotype was leaking in 5/17, insufficient clearance in 3/17, bradykinesia in 4/17, complex dysphagia in 1/17, and no dysphagia in 4/17 patients. No significant correlation was found for the FEES score and FEES severity with serum NfL and serum GFAP (Table 1). FEES severity score correlated with the Unified Multiple System Atrophy Rating Scale (UMSRARS) total score and showed a tendency to correlate with the UMSARS item dysphagia (Table 1). In the subgroup analysis of MSA‐P and MSA‐C patients, no significant correlation with serum NfL and GFAP was detected (data not shown).

**TABLE 1:** Spearman correlation analysis of dysphagia in multiple system atrophy patients (N = 17)

Parameter	*r*	*P*‐value
FEES score with		
UMSARS	0.236	0.362
UMSARS item dysphagia	0.303	0.238
NfL	−0.085	0.746
GFAP	−0.032	0.905
FEES severity score with		
UMSARS	0.575	0.016*
UMSARS item dysphagia	0.469	0.058
NfL	−0.005	0.984
GFAP	0.059	0.829

* *P* < 0.05 significant.

Abbreviations: FEES, flexible endoscopic evaluation of swallowing; GFAP, glial fibrillary acid protein in serum; NfL, neurofilament light chain in serum; UMSARS, Unified Multiple System Atrophy Rating Scale.

This is the first study investigating the association of serum biomarkers related to disease severity and FEES scores in MSA patients with blinded FEES scoring. FEES is a complex investigation, which is not available in every center. To overcome this issue, a biomarker screening approach would be desirable. However, data from the present study could not support the usability of serum NfL and GFAP as screening biomarkers for dysphagia in MSA.

The main limitation of this study is the limited sample size. Further studies investigating multiple biomarkers in a larger patient cohort of MSA patients would be of major interest in the future.

## Author Roles

(1) Research Project: A. Conception, B. Organization, C. Execution; (2) Statistical Analysis: A. Design, B. Execution, C. Review and Critique; (3) Manuscript Preparation: A. Writing of the First Draft, B. Review and Critique.

M.K.: 1A, 1B, 1C, 2A, 2B, 3A, 3B.

K.P.‐M.: 1C, 3B.

F.B.: 1C, 3B.

F.W.: 1B, 2C, 3B.

M.H.: 1C, 3B.

L.K.: 1C, 3B.

L.Y.: 1C, 2B, 3B.

C.S.: 1C, 3B.

G.U.H.: 1A, 1B, 1C, 2A, 2C, 3B.

T.W.: 1A, 1B, 1C, 2A, 2C, 3B.

## Disclosures

**Ethical Compliance Statement**: The authors confirm that they have read the Journal's position on issues involved in ethical publication and affirm that this work is consistent with those guidelines. The authors obtained the approval of local institutional review boards for this work (MHH: ID: 8666_BO_K_2019). Written informed consent was obtained from every participant in advance of study participation.

**Funding Sources and Conflicts of Interest**: The authors received no specific funding for this study. There were no conflicts of interest relevant to this study to declare. M.K. serves as a consultant for AbbVie and received honoraria for scientific presentations from AbbVie, Bial, and Ever. M.K. was funded by the German Parkinson's Disease Association, the MHH plus Foundation (Hannover, Germany), and the Petermax Müller Foundation (Hannover, Germany).

**Financial Disclosures for the Previous 12 Months**: The authors do not have any relevant disclosures concerning this publication.

## Supporting information


**Table S1.** Demographics, patient characteristics, and biomarkers.

## Data Availability

The data that support the findings of this study are available from the corresponding author upon reasonable request.
