# Supplementary material for: Dysphagia in Multiple System Atrophy Does Not Correlate with Serum Neurofilament Light Chain and Glial Fibrillary Acidic Protein
Source: Mov Disord. 2025 Oct 11;41(2):536–7. doi: 10.1002/mds.70092 (PMC12951257; doi:10.1002/mds.70092)
Supplement: Supplementary file 1 — Table S1. Demographics, patient characteristics, and biomarkers. [file MDS-41-536-s001.docx]

**Supplementary table 1. Demographics, patient characteristics and biomarker**

|  | Mean | SD | Min | max |
| --- | --- | --- | --- | --- |
| Age | 59.6 | 7.1 | 45 | 75 |
| Hoehn and Yahr stage | 3.5 | 0.8 | 2.5 | 5 |
| MDS-UPDRS III | 55.3 | 18.6 | 25 | 81 |
| UMSARS sum of part 1 and 2 | 51.9 | 14.6 | 34 | 81 |
| UMSARS item dysphagia | 1.6 | 1.0 | 0 | 3 |
| FEES Score | 14.1 | 8.0 | 2 | 35 |
| FEES severity score | 1.1 | 0.8 | 0 | 3 |
| NfL pg/mL | 30.5 | 11.1 | 13.0 | 47.6 |
| GFAP pg/mL | 118.3 | 72.2 | 45.8 | 325.3 |

Abbreviations: MDS-UPDRS III movement disorders society sponsored revision of the unified Parkinson’s disease rating scale part III; UMSARS unified multiple system atrophy rating scale; FEES flexible endoscopic evaluation of swallowing; NfL neurofilament light chain in serum; GFAP glial fibrillary acid protein in serum.
